# Prognostic Value of Serum 1,5-anhydroglucitol Levels in Patients with Acute Myocardial Infarction

**DOI:** 10.31083/j.rcm2312394

**Published:** 2022-12-02

**Authors:** Yijia Wang, Ruiyue Yang, Yanan Zhang, Zhe Wang, Xinyue Wang, Siming Wang, Wenduo Zhang, Xue Yu, Jun Dong, Wenxiang Chen, Fusui Ji

**Affiliations:** ^1^Department of Cardiology, Beijing Hospital, National Center of Gerontology; Institute of Geriatric Medicine, Chinese Academy of Medical Sciences, 100730 Beijing, China; ^2^Chinese Academy of Medical Sciences and Graduate school of Peking Union Medical College, 100730 Beijing, China; ^3^The Key Laboratory of Geriatrics, Beijing Institute of Geriatrics, Institute of Geriatric Medicine, Chinese Academy of Medical Sciences, Beijing Hospital/National Center of Gerontology of National Health Commission, 100730 Beijing, China; ^4^Department of Critical Care Medicine, The Affiliated Hospital of Qingdao University, 266000 Qingdao, Shandong, China; ^5^Department of Cardiology, China-Japan Friendship Hospital, 100029 Beijing, China

**Keywords:** acute myocardial infarction, 1,5-anhydroglucitol, major adverse cardiovascular and cerebrovascular events, all-cause mortality

## Abstract

**Background:**

Diabetes mellitus is a major risk element for cardiovascular disease. In the present study we investigated whether 1,5-anhydroglucitol (1,5-AG), a new marker for glucose monitoring, can predict patient outcome following acute myocardial infarction (AMI).

**Methods:**

A total of 270 AMI patients who underwent coronary angiography (CAG) at Beijing Hospital from March 2017 to 2020 were enrolled in this prospective cohort study. The serum 1,5-AG concentration and biochemical indicators were evaluated prior to CAG. Cox regression analysis was used to investigate the relationship between 1,5-AG levels and major adverse cardiovascular and cerebrovascular events (MACCEs), and with all-cause mortality.

**Results:**

During the median follow-up period of 44 months, 49 MACCEs occurred and 33 patients died. The 1,5-AG level was significantly lower in the MACCEs group than in the MACCEs-free group (*p* = 0.001). Kaplan-Meier analysis also revealed that low 1,5-AG levels were associated with MACCEs (*p *< 0.001) and with all-cause mortality (*p* = 0.001). Multivariate analysis showed that low 1,5-AG (≤8.8 μg/mL) was an independent predictor of MACCEs (hazard ratio (HR) 2.000, 95% confidence interval (CI): 1.047–3.821, *p* = 0.036). However, 1,5-AG was not a significant predictor for all-cause mortality in AMI patients (*p *> 0.05).

**Conclusions:**

Low 1,5-AG levels can predict MACCEs in AMI patients, but not all-cause mortality.

**Clinical Trial Registration:**

NCT03072797.

## 1. Introduction

Acute myocardial infarction (AMI) is a major cause of death and disability worldwide [1]. Patients who survive AMI remain at high risk of heart failure, recurrent AMI, and stroke even with revascularization and secondary prevention drug therapy [2, 3, 4]. Several promising biomarkers have recently been suggested to improve the risk stratification of AMI patients.

1,5-Anhydroglucitol (1,5-AG) is a new biomarker of acute hyperglycemia in cardiac diabetology, reflecting glucose status within 1–3 days or 2 weeks [5, 6, 7]. Glucose abnormalities are very common comorbidities in cardiovascular disease patients, and approximately 50%–70% of patients with coronary artery disease (CAD) have impaired glycemic regulation [8, 9]. Some studies have reported that glycemia fluctuation may play a major role in the development of atherosclerosis and could also be an independent predictor for cardiovascular comorbidities in patients with diabetes [10, 11]. However, it remains unclear whether acute glycemic fluctuations have any prognostic significance in patients with AMI.

1,5-AG is a carbon-1 deoxy pyranose and is primarily obtained from dietary intake. It is metabolically inert and present at almost constant levels in blood and tissues [12]. Glucose is structurally similar to 1,5-AG. When blood glucose levels rise sharply, high urinary glucose levels prevent the reabsorption of 1,5-AG by renal tubules, thereby resulting in 1,5-AG excretion from the urine and decreased serum levels [13]. Previous studies demonstrated that low 1,5-AG concentrations were associated with an increased risk of cardiovascular events in patients without CAD or stroke [14, 15]. However, it is still unclear whether serum 1,5-AG is useful for predicting major adverse cardiovascular and cerebrovascular events (MACCEs) in patients with AMI. The aim of this study was therefore to examine the prognostic significance of acute glucose fluctuations in AMI patients, as reflected by 1,5-AG levels.

## 2. Methods

### 2.1 Protocol Design and Populations

All patients in this prospective cohort study were from the Beijing Hospital Atherosclerosis Study (BHAS) (Clinicaltrials.gov registry number NCT03072797). Included were AMI patients who received coronary angiography (CAG) from March 2017 to 2020 at Beijing Hospital. Exclusion criteria were severe cardiac insufficiency, severe valvular heart disease, severe pulmonary disease malignancy or primary pulmonary hypertension, and severe hepatic or renal impairment. The study protocol obeyed the principles of the Declaration of Helsinki and was approved by the Ethics Committee of Beijing Hospital (2016BJYYEC-121-02). All patients provided written informed consent.

### 2.2 Clinical and Laboratory Data

Data for various demographic parameters were collected from hospital records. The serum 1,5-AG level was measured in all patients before their CAG by KingMed Diagnostics on a Roche Modular 702 system using a pyranose oxidase assay kit (batch number: 20-0825) from Beijing Strong Biotech. Glucose, total cholesterol (TC), triglycerides (TG), low-density lipoprotein cholesterol (LDL-C), high-density lipoprotein cholesterol (HDL-C), and creatinine (Crea) levels were evaluated in the clinical laboratory of Beijing Hospital using assay kits (batch number are 846RJR, 841RAS, 933RAS, 846RBS, 843RAS, 850RKR respectively) from Sekisui Medical Technologies (Osaka, Japan) on a Hitachi 7180 chemistry analyzer. All laboratory parameters were measured in venous blood samples collected within 24 hours of patient admission and prior to CAG.

### 2.3 AMI Definition and Gensini Score

AMI was defined as recommended in the current guidelines [16]. The complexity of coronary artery stenosis is reflected in the Gensini score [17], which was determined in all AMI patients by giving a score to each coronary stenosis. A higher Gensini score indicates more severe coronary artery stenosis.

### 2.4 Outcome and Follow-Up

The primary endpoint was the composite endpoints of MACCEs. These were comprised of cardiac death, non-fatal myocardial infarction, stroke, revascularization, and re-hospitalization for other cardiovascular causes. The secondary endpoint was all-cause mortality. All patients were monitored annually, with adverse incidents recorded at each visit.

### 2.5 Statistical Analysis

Continuous variables were described as the mean ± standard deviation, or medians (Q1, Q3 quartiles). Comparisons between groups were performed using the Student’s *t*-test or Mann-Whitney U-test, depending on whether the data was normally distributed. Categorical variables were expressed as numbers (percentages), and comparisons between two groups were made using the Chi-square test. Univariate and multivariate Cox proportional hazard models were used to analyze the association between low 1,5-AG levels and MACCEs. Variables with statistically significant relevance (*p*-value < 0.05) in the univariate Cox regression analysis model were included in the multivariate analysis model. These included age, estimated glomerular filtration rate (eGFR), Gensini score, glucose, 1,5-AG, and diabetes mellitus (DM). Sensitivity analysis was performed using 1,5-AG at the optimal threshold value (8.8 μg/mL), as determined by the receiver operating characteristic curve (ROC). The hazard ratio (HR) was determined for quintiles of 1,5-AG concentrations. Kaplan-Meier analysis was used to compute the time to MACCEs in AMI patients stratified by 1,5-AG concentration (≤8.8 μg/mL and >8.8 μg/mL). Survival curves were compared using the log-rank test. All statistical analyses were performed using SPSS version 26.0 (IBM, Chicago, IL, USA).

## 3. Results

### 3.1 Baseline Characteristics

**Fig. 1.**
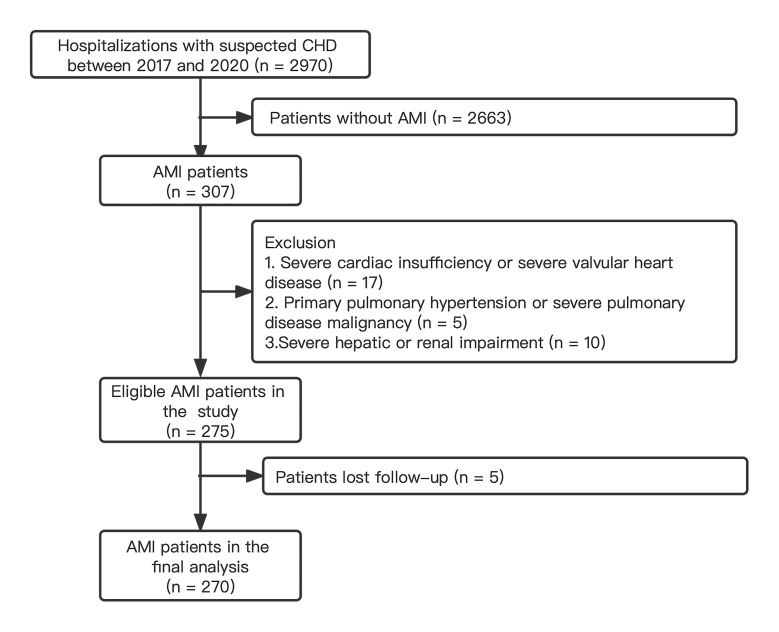
**Patient flow chart for the study cohort**. Abbreviations: AMI, acute myocardial infarction; CHD, coronary heart disease.

A total of 270 hospitalized patients were investigated (Fig. 1). The average age of this study cohort was 67.7 ± 11.8 years, with 188 (69.6%) patients being men. Patients with MACCEs were older, had higher blood glucose levels and Gensini scores, a higher proportion of combined DM, and a lower eGFR compared to patients without MACCEs (all *p *< 0.05). They also had significantly lower levels of 1,5-AG than patients without MACCEs [9.65 (3.29, 21.69) μg/mL *vs*. 18.88 (8.94, 28.74) μg/mL, respectively, *p* = 0.001] (Table 1). Similarly, the 1,5-AG concentration in patients who died of any cause was lower than that of patients who were alive at the end of the study period [6.91 (2.96, 19.90) μg/mL *vs*. 18.22 (7.93, 28.34) μg/mL, respectively, *p* = 0.009]. Patients were divided into two groups according to the ROC cut-off value (8.8 μg/mL) and their basic clinical information was compared. Significant differences between the low and high 1,5-AG groups were observed for glucose, DM history, gender, eGFR, and Gensini score (**Supplementary Table 1**).

**Table 1.** **Baseline characteristics of patients with and without MACCEs**.

Variable	MACCEs-free group	MACCEs group	*p*-value
	(n = 221)	(n = 49)	
Age, years	66.8 ± 11.9	71.3 ± 11.9	0.011
Male gender, %	154 (70.0%)	34 (69.4%)	0.933
BMI, ${\mathrm{kg/m}}^{2}$	25.0 (22.9, 26.9)	24.6 (23.0, 26.6)	0.764
SBP, mmHg	133 ± 21	134 ± 22	0.778
DBP, mmHg	76 ± 10	74 ± 13	0.684
Hypertension, %	150 (67.9%)	38 (77.6%)	0.183
Dyslipidemia, %	74 (33.5%)	23 (46.9%)	0.076
Diabetes mellitus, %	126 (57.0%)	40 (81.6%)	0.001
Stroke, %	17 (7.7%)	6 (12.2%)	0.302
Family history of early onset CAD, %	21 (9.5%)	2 (4.1%)	0.219
Smoking, %	80 (36.2%)	13 (26.5%)	0.198
Glucose, mmol/L	6.80 (5.60, 8.95)	8.65 (6.13, 10.48)	0.006
1,5-AG, µg/mL	18.88 (8.94, 28.74)	9.65 (3.29, 21.69)	0.001
TC, mmol/L	3.66 (3.04, 4.43)	3.46 (2.93, 4.02)	0.121
TG, mmol/L	1.48 (1.10, 1.96)	1.51 (1.11, 1.74)	0.603
HDL-C, mmol/L	0.92 (0.78, 1.11)	0.86 (0.75, 1.10)	0.150
LDL-C, mmol/L	2.17 (1.68, 2.79)	2.06 (1.53, 2.61)	0.248
Crea, µmol/L	82.0 (72.0, 96.0)	89.0 (75.5, 112.0)	0.055
eGFR, mL/min/1.73 $m^{2}$	74.53 (55.97, 94.05)	59.88 (38.51, 82.53)	0.007
Gensini score	44.00 (25.75, 74.50)	60.00 (32.50, 101.00)	0.019
Type of myocardial infarction			0.417
NSTEMI, %	154 (69.7%)	37 (75.5%)	
STEMI, %	67 (30.3%)	12 (24.5%)	

Abbreviations: MACCEs, major adverse cardiovascular and cerebrovascular events; BMI, body mass index; SBP, systolic blood pressure; DBP, diastolic blood pressure; TC, triglycerides; TG, total cholesterol; LDL-C, low-density lipoprotein cholesterol; HDL-C, high-density lipoprotein cholesterol; Crea, creatinine; eGFR, estimated glomerular filtration rate; NSTEMI, Non-ST Elevation Myocardial Infarction; STEMI, ST Elevation Myocardial Infarction.

### 3.2 Predictive Value of 1,5-AG for MACCEs and Mortality in AMI Patients

**Fig. 2.**
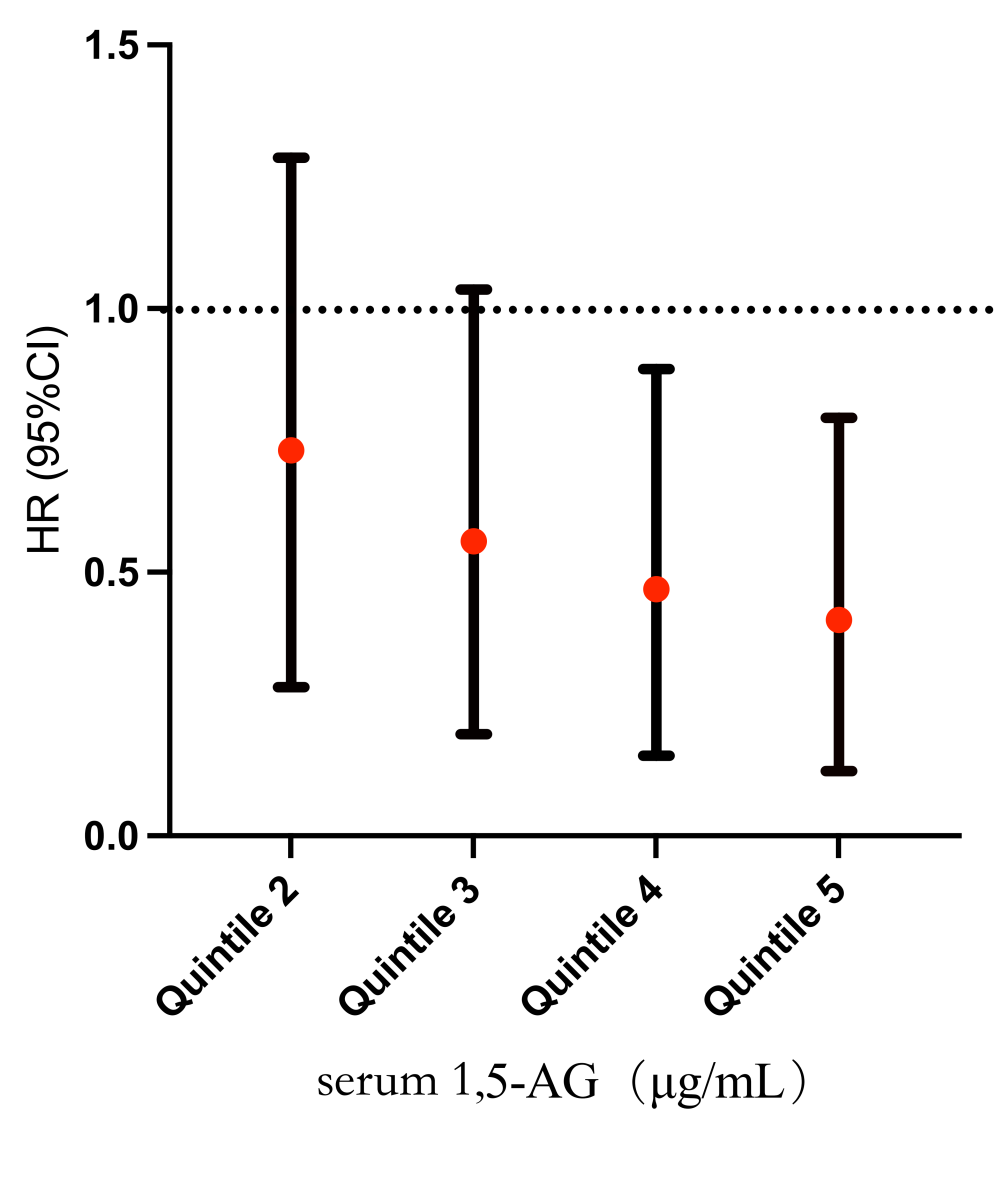
**Hazard ratio for MACCEs according to 1,5-AG quintiles**. The vertical lines indicate the hazard ratio and 95% CI for quintiles 2 to 5 of 1,5-AG relative to quintile 1. Quintile 1 (1,5-AG <4.88 μg/mL); Quintile 2 (4.88 ≤ 1,5-AG < 12.93 μg/mL); Quintile 3 (12.93 ≤ 1,5-AG < 21.00 μg/mL); Quintile 4 (21.00 ≤ 1,5-AG < 30.35 μg/mL); Quintile 5 (1,5-AG ≥30.35 μg/mL). Abbreviations: 1,5-AG, 1,5-anhydroglucitol; HR, hazard ratio.

Overall, 49 (18.1%) MACCEs occurred during the median follow-up period of 44.0 (33.8, 54.0) months. These included 18 cardiac deaths, 1 myocardial infarction, 11 revascularizations, 2 strokes, and 17 re-hospitalizations for other cardiac reasons. Relative to patients in the first quintile, the HR for MACCEs was significantly lower in patients with a 1,5-AG level above the fourth quintile (1,5-AG ≥21.0 μg/mL). The fourth quintile HR was 0.367 (95% confidence interval (CI): 0.152–0.885, *p* = 0.026), while the fifth quintile HR was 0.312 (95% CI: 0.123–0.793, *p* = 0.014) (Fig. 2).

**Fig. 3.**
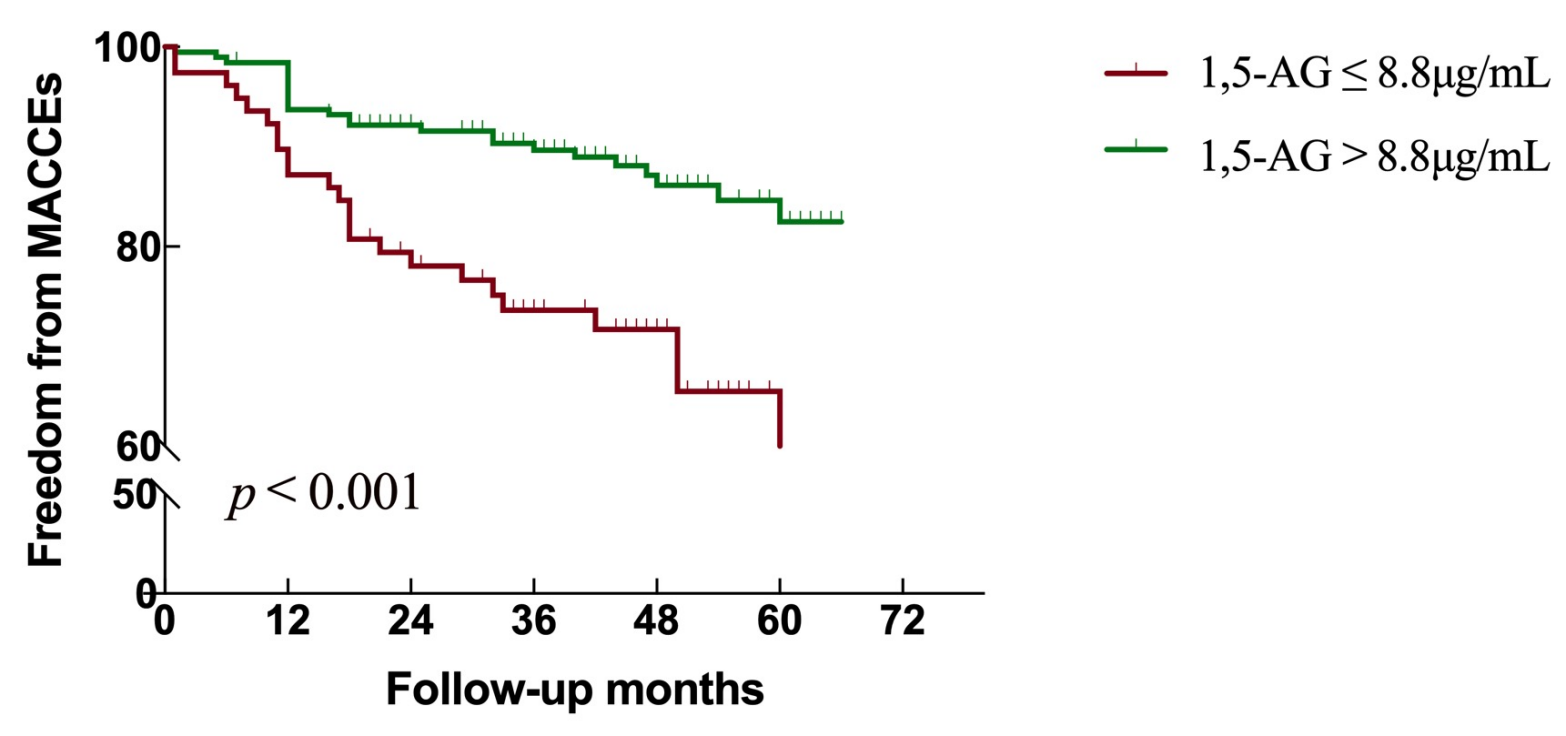
**Kaplan-Meier plot of 1,5-AG to predict the occurrence of MACCEs in AMI patients**. 1,5-AG as a categorical value was used to predict MACCEs according to the cut-off value (8.8 μg/mL). Abbreviations: 1,5-AG, 1,5-anhydroglucitol; MACCEs, major adverse cardiovascular and cerebrovascular events.

**Fig. 4.**
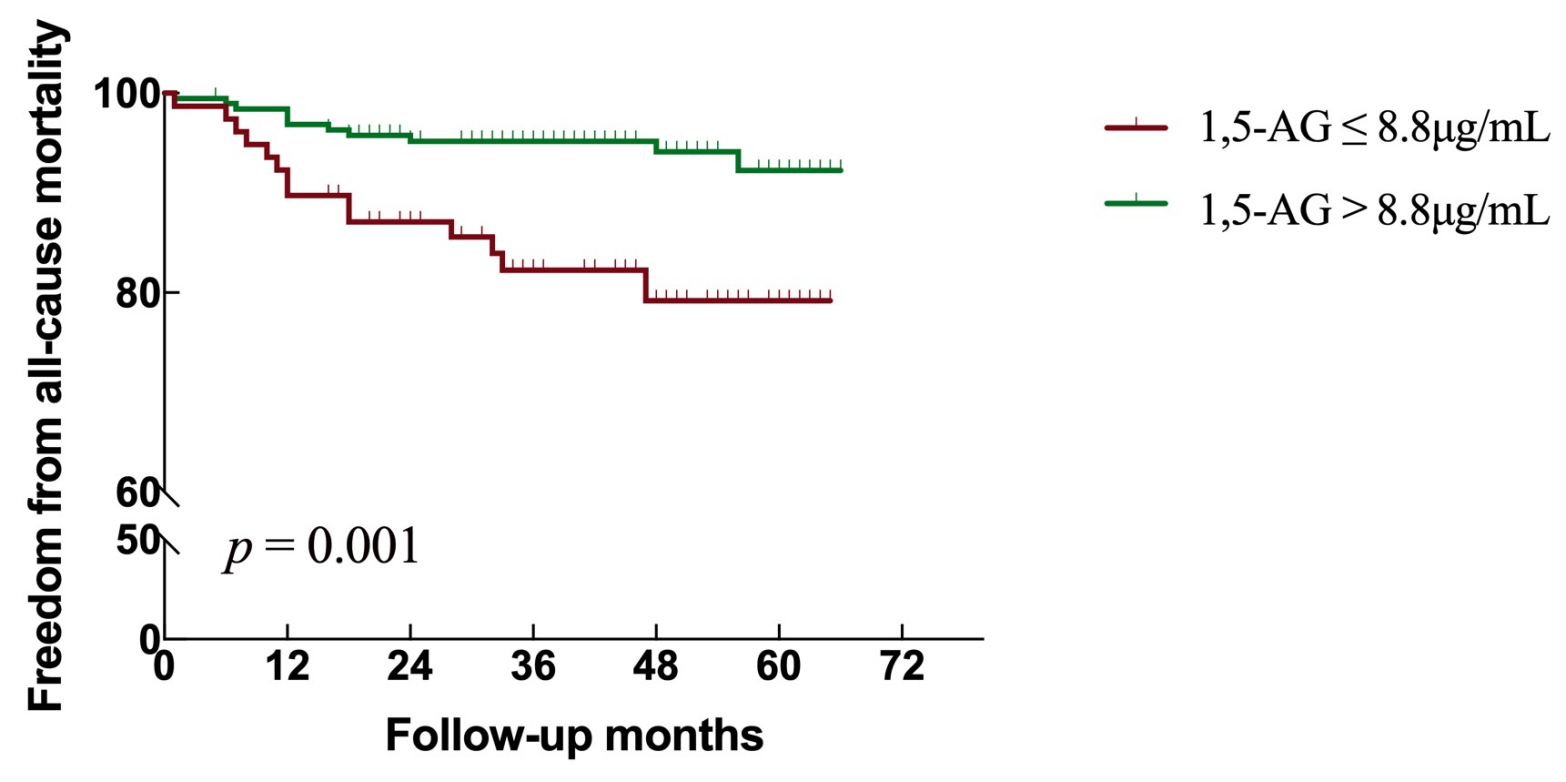
**Kaplan-Meier plot of 1,5-AG to predict all-cause mortality in AMI patients**. 1,5-AG as a categorical value was used to predict all-cause mortality according to the cut-off value (8.8 μg/mL). Abbreviations: 1,5-AG, 1,5-anhydroglucitol.

After a median follow-up time of 44 months, Kaplan-Meier analysis revealed the cumulative event rate for MACCEs was 24 (30.8%) in the low 1,5-AG group (≤8.8 μg/mL) and 25 (13.0%) in the high 1,5-AG group (1,5-AG >8.8 μg/mL) (*p *< 0.001, Fig. 3). A total of 33 patients died (all causes), with an incidence rates of 17.9% in low 1,5-AG patients and 5.7% in high 1,5-AG patients (*p* = 0.001, Fig. 4). Therefore, the incidence of MACCEs and of all-cause mortality were both significantly higher in AMI patients with a low level of 1,5-AG.

Univariate analysis showed that age, eGFR, Gensini score, glucose, DM and 1,5-AG were significant risk factors for MACCEs in AMI patients (all *p *< 0.05, Table 2). The serum 1,5-AG level of ≤8.8 μg/mL was associated with an increased risk of MACCEs (HR = 2.718, 95% CI: 1.551–4.765, *p* = 0.001) (Table 2). Using 8.8 μg/mL as the threshold, multivariate Cox regression analysis showed that low 1,5-AG was associated with a significantly increased risk of MACCEs (HR = 2.000, 95% CI: 1.047–3.821, *p* = 0.036) (Table 3). However, the 1,5-AG level was not an independent predictor of all-cause mortality in patients with AMI (*p *> 0.05).

**Table 2.** **Univariate COX regression analysis for MACCEs in the overall cohort**.

Variable	HR	95% CI	*p*-value
Age, years	1.033	1.007–1.060	0.014
Male gender, %	1.005	0.547–1.844	0.988
BMI, ${\mathrm{kg/m}}^{2}$	0.988	0.905–1.079	0.791
SBP, mmHg	0.998	0.985–1.011	0.760
DBP, mmHg	1.003	0.976–1.031	0.840
Hypertension, %	1.526	0.780–2.987	0.217
Dyslipidemia, %	1.502	0.857–2.634	0.156
Diabetes mellitus, %	3.008	1.497–6.369	0.002
Stroke, %	1.523	0.648–3.580	0.334
Family history of premature cardiovascular disease, %	0.533	0.129–2.197	0.384
Smoking, %	1.433	0.760–2.703	0.266
Glucose, mmol/L	1.083	1.012–1.159	0.021
Categorical variables			
1,5-AG >8.8 µg/mL	reference	reference	reference
1,5-AG ≤8.8 µg/mL	2.718	1.551–4.765	0.001
TC, mmol/L	0.772	0.552–1.078	0.129
TG, mmol/L	0.841	0.591–1.197	0.337
HDL-C, mmol/L	0.328	0.089–1.213	0.095
LDL-C, mmol/L	0.813	0.555–1.190	0.287
Crea, µmol/L	1.001	1.000–1.003	0.105
eGFR, mL/min/1.73 $m^{2}$	0.987	0.978–0.997	0.012
Gensini score	1.010	1.003–1.017	0.003
Type of myocardial infarction			
NSTEMI, %	reference	reference	reference
STEMI, %	0.762	0.397–1.462	0.414

Abbreviations: MACCEs, major adverse cardiovascular and cerebrovascular events; BMI, body mass index; SBP, systolic blood pressure; DBP, diastolic blood pressure; 1,5-AG, 1,5-anhydroglucitol; TC, triglycerides; TG, total cholesterol; HDL-C, high-density lipoprotein cholesterol; LDL-C, low-density lipoprotein cholesterol; Crea, creatinine; eGFR, estimated glomerular filtration rate; NSTEMI, Non-ST Elevation Myocardial Infarction; STEMI, ST Elevation Myocardial Infarction.

**Table 3.** ** Multivariate analysis with MACCEs as the endpoint event**.

Variable	HR	95% CI	*p*-value
Age, years	1.011	0.981–1.043	0.471
Glucose	0.984	0.893–1.085	0.751
eGFR, mL/min/1.73 $m^{2}$	0.995	0.984–1.007	0.418
Gensini score	1.007	0.999–1.014	0.069
Diabetes mellitus,%	1.882	0.795–4.457	0.151
Categorical variables			
1,5-AG >8.8 µg/mL	reference	reference	reference
1,5-AG ≤8.8 µg/mL	2.000	1.047–3.821	0.036

Multivariate model: 1,5-AG as a categorical variable, Age, Glucose, eGFR, Gensini score, Diabetes mellitus. Abbreviations: MACCEs, major adverse cardiovascular and cerebrovascular events; 1,5-AG, 1,5-anhydroglucitol; eGFR, estimated glomerular filtration rate.

## 4. Discussion

The present study found that 1,5-AG levels could predict MACCEs in AMI patients. Although we did not observe a significant association between 1,5-AG levels and all-cause mortality in these patients, the present results suggest that 1,5-AG may help to identify patients who are at increased risk of MACCEs.

Traditional cardiovascular risk factors do not accurately estimate the variation in cardiovascular risk between individuals, with most people having only one or none of the classical cardiovascular risk factors [18]. It is therefore very important to identify new risk factors. The serum 1,5-AG marker reflects acute blood glucose elevation and as such is a new indicator for diabetes management. When the glucose level exceeds the renal threshold for urine sugar, 1,5-AG is excreted through the urine, resulting in a rapid decline in serum levels [19]. Low 1,5-AG levels are therefore relevant to poor glycemic management. Due to the inverse correlation between 1,5-AG levels and blood glucose, the presence of low 1,5-AG levels during AMI may be a manifestation of acute hyperglycemia. The latter has been associated with poor prognosis, regardless of the presence of diabetes [20]. This is also consistent with the results of our recent cross-sectional study that demonstrated a non-linear relationship between low serum 1,5-AG levels and CAD severity [21]. In the present study, we found that low 1,5-AG levels also predicted the risk of medium to long-term MACCEs in AMI patients (Fig. 3). Low 1,5-AG levels should therefore be considered not just as a marker of acute phase severity, but also as a marker of persistent cardiovascular risk.

Several previous studies have shown that low 1,5-AG levels were associated with an increased risk of CAD. Ikeda *et al*. [22] found that low 1,5-AG levels were an independent predictor of CAD risk. Selvin *et al*. [23] reported a markedly increased risk of CAD, stroke, heart failure, and death in patients with a 1,5-AG level of <6.0 μg/mL and with DM (*p *< 0.05). Watanabe *et al*. [24] conducted a cohort study on 2095 healthy individuals with no CAD or stroke and with 11 years of follow-up. They found the HR for CAD in patients with a low 1,5-AG level (<14.0 μg/mL) was 2.22-fold higher than in those with high 1,5-AG (>24.5 μg/mL).

So far, however, there is a lack of data on the prognostic utility of 1,5-AG for AMI patients. In patients without CAD and DM, Ikeda *et al*. [25] found that the risk of MACCEs was 2.34-fold higher in those with <10.0 μg/mL of 1,5-AG compared to patients with ≥10.0 μg/mL. However, the study population in the present investigation was hospitalized AMI patients. These patients had been sufficiently risk stratified compared to low-risk populations, allowing us to explore biomarkers in a somewhat smaller population with shorter follow-up. Moreover, these subjects had a relatively high risk of coronary events during the follow-up period [26]. The present study found that low 1,5-AG levels were predictive of MACCEs in AMI patients. This result agrees with the findings of a recent study by Ouchi *et al*. [27], who reported that low 1,5-AG levels were predictive of long-term cardiac mortality events in acute coronary syndrome (ACS) patients.

The primary mechanism of AMI is known to be thrombosis secondary to unstable plaque rupture or intimal erosion. A recent study of 144 ACS patients with concomitant DM found that low 1,5-AG levels were an independent determinant of plaque rupture [28]. We speculate that 1,5-AG may be involved in the development of AMI, and the pathogenesis could be explained by the following considerations. First, it has been shown that 1,5-AG is closely associated with oxidative stress in DM patients, and this is known to play a crucial role in the development of atherosclerotic plaques [29, 30, 31]. Second, Teraguchi *et al*. [32] found that short-term glucose fluctuations were significantly and positively correlated with CD14+CD16+ monocytes. The receptors for these cells can be activated by binding to chemokines produced at the site of inflammation, secretion of various pro-inflammatory factors, and enhancement of the local inflammatory response. In addition, 1,5-AG may also be involved in endothelial dysfunction [33]. Monitoring of 1,5-AG levels could therefore be useful for determining the risk of coronary plaque rupture at an early stage.

## 5. Limitations of the Study

This study has several limitations. Firstly, it was a single-center study with a small population, and all patients were from the same region, thus limiting the generalizability of the results. Secondly, the use of sodium-glucose cotransporter inhibitors can affect 1,5-AG levels, and the effect of this class of drugs on the study outcomes were not considered. Furthermore, additional baseline clinical data such as the incidence of cardiogenic shock, infarct type (anterior, inferior or posterior infarction), time from symptom onset to revascularization, and the GRACE risk score need to be further refined and included in our model. Finally, further studies are required to determine whether intervention to alter the 1,5-AG concentration can reduce the risk of AMI and the incidence of MACCEs.

## 6. Conclusions

The 1,5-AG concentration is a marker of short-term blood glucose fluctuation. In the current study the 1,5-AG level was also found to be predictive of MACCEs in patients with AMI. In addition to being a possible new marker for glucose monitoring, 1,5-AG may be helpful for risk stratification of AMI patients.

## Data Availability

The datasets generated and analyzed during the current study are not publicly available due to privacy or ethical restrictions, but are available from the corresponding author on reasonable request.

## Abbreviations

1,5-AG, 1,5-anhydroglucitol; AMI, acute myocardial infarction; CAG, coronary, angiography; MACCEs, major adverse cardiovascular and cerebrovascular events; HR, hazard ratio; CI, confidence interval; CAD, Coronary artery disease; BMI, body mass index; SBP, systolic blood pressure; DBP, diastolic blood pressure; TC, total cholesterol; TG, triglycerides; HDL-C, high-density lipoprotein cholesterol; LDL-C, low-density lipoprotein cholesterol; Crea, creatinine; UA, uric acid; eGFR, estimated glomerular filtration rate; DM, diabetes mellitus.

## Supplementary Material

Supplementary material associated with this article can be found, in the online version, at https://doi.org/10.31083/j.rcm2312394.

## References

[1] Go AS, Mozaffarian D, Roger VL, Benjamin EJ, Berry JD, Borden WB (2013). Heart disease and stroke statistics–2013 update: a report from the American Heart Association. *Circulation*.

[2] Chu CY, Lin TH, Lai WT (2017). The Management and Prognostic Factors of Acute Coronary Syndrome: Evidence from the Taiwan Acute Coronary Syndrome Full Spectrum Registry. *Acta Cardiological Sinica*.

[3] Eisen A, Giugliano RP, Braunwald E (2016). Updates on Acute Coronary Syndrome: A Review. *JAMA Cardiology*.

[4] Jenča D, Melenovský V, Stehlik J, Staněk V, Kettner J, Kautzner J (2021). Heart failure after myocardial infarction: incidence and predictors. *ESC Heart Failure*.

[5] Dungan KM, Buse JB, Largay J, Kelly MM, Button EA, Kato S (2006). 1,5-Anhydroglucitol and Postprandial Hyperglycemia as Measured by Continuous Glucose Monitoring System in Moderately Controlled Patients with Diabetes. *Diabetes Care*.

[6] Kiniwa N, Okumiya T, Tokuhiro S, Matsumura Y, Matsui H, Koga M (2019). Hemolysis causes a decrease in HbA1c level but not in glycated albumin or 1,5-anhydroglucitol level. *Scandinavian Journal of Clinical and Laboratory Investigation*.

[7] Cavalot F, Pagliarino A, Valle M, Di Martino L, Bonomo K, Massucco P (2011). Postprandial Blood Glucose Predicts Cardiovascular Events and all-Cause Mortality in Type 2 Diabetes in a 14-Year Follow-up: lessons from the San Luigi Gonzaga Diabetes Study. *Diabetes Care*.

[8] Bartnik M, Rydén L, Ferrari R, Malmberg K, Pyörälä K, Simoons M (2004). The prevalence of abnormal glucose regulation in patients with coronary artery disease across Europe. The Euro Heart Survey on diabetes and the heart. *European Heart Journal*.

[9] Bartnik M, Ryden L, Malmberg K, Ohrvik J, Pyorala K, Standl E (2007). Oral glucose tolerance test is needed for appropriate classification of glucose regulation in patients with coronary artery disease: a report from the Euro Heart Survey on Diabetes and the Heart. *Heart*.

[10] Hu Y, Liu W, Huang R, Zhang X (2010). Postchallenge plasma glucose excursions, carotid intima-media thickness, and risk factors for atherosclerosis in Chinese population with type 2 diabetes. *Atherosclerosis*.

[11] Su G, Mi S, Tao H, Li Z, Yang H, Zheng H (2011). Association of glycemic variability and the presence and severity of coronary artery disease in patients with type 2 diabetes. *Cardiovascular Diabetology*.

[12] Buse JB, Freeman JL, Edelman SV, Jovanovic L, McGill JB (2003). Serum 1,5- anhydroglucitol (GlycoMarkTM): A short-term glycemic marker. *Diabetes Technology & Therapeutics*.

[13] Yamanouchi T, Akanuma Y (1994). Serum 1,5-anhydroglucitol (1,5 AG): New clinical marker for glycemic control. *Diabetes Research and Clinical Practice*.

[14] Fujiwara T, Yoshida M, Akashi N, Yamada H, Tsukui T, Nakamura T (2016). Lower 1,5-anhydroglucitol is associated with adverse clinical events after percutaneous coronary intervention. *Heart and Vessels*.

[15] Ikeda N, Hara H, Hiroi Y (2014). 1,5-Anhydro-d-glucitol predicts coronary artery disease prevalence and complexity. *Journal of Cardiology*.

[16] Thygesen K, Alpert JS, Jaffe AS, Chaitman BR, Bax JJ, Morrow DA (2018). Fourth Universal Definition of Myocardial Infarction (2018). *Journal of the American College of Cardiology*.

[17] Gensini GG (1983). A more meaningful scoring system for determining the severity of coronary heart disease. *The American Journal of Cardiology*.

[18] Khot UN, Khot MB, Bajzer CT, Sapp SK, Ohman EM, Brener SJ (2003). Prevalence of conventional risk factors in patients with coronary heart disease. *Japan Automobile Manufacturers Association*.

[19] Akanuma Y, Morita M, Fukuzawa N, Yamanouchi T, Akanuma H (1988). Urinary excretion of 1,5-anhydro-D-glucitol accompanying glucose excretion in diabetic patients. *Diabetologia*.

[20] Upur H, Li JL, Zou XG, Hu YY, Yang HY, Abudoureyimu A (2022). Short and long-term prognosis of admission hyperglycemia in patients with and without diabetes after acute myocardial infarction: a retrospective cohort study. *Cardiovascular Diabetology*.

[21] Yang R, Zhang W, Wang X, Wang S, Zhou Q, Li H (2022). Nonlinear association of 1,5-anhydroglucitol with the prevalence and severity of coronary artery disease in Chinese patients undergoing coronary angiography. *Frontiers in Endocrinology*.

[22] Ikeda N, Hara H, Hiroi Y (2015). Ability of 1,5-Anhydro-d-glucitol Values to Predict Coronary Artery Disease in a Non-Diabetic Population. *International Heart Journal*.

[23] Selvin E, Rawlings A, Lutsey P, Maruthur N, Pankow JS, Steffes M (2016). Association of 1,5-Anhydroglucitol with Cardiovascular Disease and Mortality. *Diabetes*.

[24] Watanabe M, Kokubo Y, Higashiyama A, Ono Y, Miyamoto Y, Okamura T (2011). Serum 1,5-anhydro-d-glucitol levels predict first-ever cardiovascular disease: an 11-year population-based Cohort study in Japan, the Suita study. *Atherosclerosis*.

[25] Ikeda N, Hara H, Hiroi Y, Nakamura M (2016). Impact of serum 1,5-anhydro-d-glucitol level on prediction of major adverse cardiac and cerebrovascular events in non-diabetic patients without coronary artery disease. *Atherosclerosis*.

[26] Zhang W, Yang R, Yu X, Wang S, Wang X, Mu H (2022). Design, methods and baseline characteristics of the Beijing Hospital Atherosclerosis Study: a prospective dynamic cohort study. *Annals of Translational Medicine*.

[27] Ouchi S, Shimada K, Miyazaki T, Takahashi S, Sugita Y, Shimizu M (2017). Low 1,5-anhydroglucitol levels are associated with long-term cardiac mortality in acute coronary syndrome patients with hemoglobin a1c levels less than 7.0. *Cardiovascular Diabetology*.

[28] Su G, Gao M, Shi G, Dai X, Yao W, Zhang T (2020). Effect of 1,5-anhydroglucitol levels on culprit plaque rupture in diabetic patients with acute coronary syndrome. *Cardiovascular Diabetology*.

[29] Pignatelli P, Menichelli D, Pastori D, Violi F (2018). Oxidative stress and cardiovascular disease: new insights. *Kardiologia Polska*.

[30] Kim MJ, Jung HS, Hwang-Bo Y, Cho SW, Jang HC, Kim SY (2013). Evaluation of 1,5-anhydroglucitol as a marker for glycemic variability in patients with type 2 diabetes mellitus. *Acta Diabetologica*.

[31] Kohata Y, Ohara M, Nagaike H, Fujikawa T, Osaka N, Goto S (2020). Association of Hemoglobin a1c, 1,5-Anhydro-d-Glucitol and Glycated Albumin with Oxidative Stress in Type 2 Diabetes Mellitus Patients: A Cross-Sectional Study. *Diabetes Therapy*.

[32] Teraguchi I, Imanishi T, Ozaki Y, Tanimoto T, Orii M, Shiono Y (2014). Impact of glucose fluctuation and monocyte subsets on coronary plaque rupture. *Nutrition, Metabolism and Cardiovascular Diseases*.

[33] Morishita T, Uzui H, Mitsuke Y, Tada H (2022). Prognostic value of 1,5-anhydro-D-glucitol incorporating syntax score in acute coronary syndrome. *Heart and Vessels*.

