# Supplementary material for: Prognostic Value of Serum 1,5-anhydroglucitol Levels in Patients with Acute Myocardial Infarction
Source: Rev Cardiovasc Med. 2022 Dec 2;23(12):394. doi: 10.31083/j.rcm2312394 (PMC11270454; doi:10.31083/j.rcm2312394)
Supplement: Supplementary file 1 [file 2153-8174-23-12-394-s1.docx]

**Supplemental Table 1. Baseline demographics and clinical characteristics.**

| Variable | All  （n=270） | Group1  1,5-AG≤8.8μg/ml  （n=78） | Group2  1,5-AG＞8.8μg/ml  （n=192） | *p*-value |
| --- | --- | --- | --- | --- |
| Age, years | 67.7±11.8 | 69.6±12.1 | 66.7±11.9 | 0.850 |
| Male gender, % | 188(69.6%) | 47(60.3%) | 141(73.4%) | 0.033 |
| BMI, kg/m2 | 24.9(22.9,26.8) | 24.8(23.1,28.2) | 24.9(22.7,26.6) | 0.229 |
| Smoking, % | 93(34.4%) | 22(28.2%) | 71(37.0%) | 0.169 |
| Hypertension, % | 188(69.6%) | 58(74.4%) | 130(67.7%) | 0.281 |
| Diabetes mellitus, % | 166(61.5%) | 74(94.9%) | 92(47.9%) | ＜0.001 |
| Dyslipidemia, % | 97(35.9%) | 29(37.2%) | 68(35.4%) | 0.784 |
| Stroke, % | 23(8.5%) | 8(10.3%) | 15(7.8%) | 0.514 |
| Family history of early onset CAD, % | 23(8.5%) | 6(7.7%） | 17(8.9%) | 0.757 |
| Glucose, mmol/L | 7.40(5.78,9.70) | 9.50(7.30,12.98) | 6.35(5.43,8.08) | ＜0.001 |
| TC, mmol/L | 3.52(2.99,4.28) | 3.52(2.99,4.39) | 3.75(3.02,4.41) | 0.482 |
| TG, mmol/L | 1.50(1.13,1.93) | 1.50(1.19,1.84) | 1.49(1.09,1.97) | 0.785 |
| HDL-C, mmol/L | 0.89(0.78,1.05) | 0.86(0.76,1.02) | 0.92(0.78,1.11) | 0.176 |
| LDL-C, mmol/L | 2.08(1.63,2.72) | 2.06(1.65,2.60) | 2.19(1.63,2.79) | 0.678 |
| Crea, umol/L | 83.0(74.0,100.0) | 83.00(72.00,112.00) | 82.00(73.25,94.75) | 0.321 |
| eGFR, ml/min/1.73m^2^ | 72.33(52.05,93.26) | 63.30(41.21,84.25) | 75.61(56.98,93.98) | 0.006 |
| Gensini score | 50.00(28.50,79.25) | 52.25(33.75,93.13) | 43.50(24.13,74.88) | 0.020 |

Note: Continuous data are presented as means ± standard deviation (SD) or median (inter-quartile range), and categorical data were shown as n (%).

Abbreviations: 1,5-AG, 1,5-anhydroglucitol; BMI, body mass index; SBP, systolic blood pressure; DBP, diastolic blood pressure; TC, triglycerides; TG, total cholesterol; HDL-C, high-density lipoprotein cholesterol; LDL-C, low-density lipoprotein cholesterol; Crea, creatinine; eGFR, estimated glomerular filtration rate.
